# Nonmotor Symptoms in Early- and Advanced-Stage Parkinson's Disease Patients on Dopaminergic Therapy: How Do They Correlate with Quality of Life?

**DOI:** 10.1155/2014/587302

**Published:** 2014-03-06

**Authors:** Peter Valkovic, Jan Harsany, Marta Hanakova, Jana Martinkova, Jan Benetin

**Affiliations:** ^1^Second Department of Neurology, Faculty of Medicine, Comenius University, Limbova 5, 833 05 Bratislava, Slovakia; ^2^Laboratory of Motor Control, Institute of Normal and Pathological Physiology, Slovak Academy of Sciences, Sienkiewiczova 1, 813 71 Bratislava, Slovakia; ^3^Department of Pharmacology and Toxicology, Faculty of Pharmacy, Comenius University, Odbojárov 10, 832 32 Bratislava, Slovakia; ^4^Department of Neurology, Faculty of Medicine, Slovak Medical University, Ruzinovska 6, 826 06 Bratislava, Slovakia

## Abstract

To determine the impact of nonmotor symptoms (NMS) on health-related quality of life (HRQoL) we examined 100 Parkinson's disease (PD) patients on dopaminergic medications. An “early-stage” (ES) and an “advanced-stage” (AS) groups were formed. HRQoL was established by the questionnaire PDQ-8, number of NMS by NMSQuest, and severity and frequency of NMS by the assessment scale NMSS. The total NMS averaged 11.3 (ES = 9.6, AS = 12.8). The NMSS domain correlation profiles for ES and AS did not fundamentally differ; however, the domains attention/memory and mood/apathy correlated moderately to strongly with HRQoL in ES, while the sleep/fatigue domain correlated moderately with HRQoL in AS. Weakly correlating domains were sleep/fatigue in ES and cardiovascular, attention/memory, and mood/apathy domains in AS. In view of these findings we strongly recommend systematic, active screening and therapy for neuropsychiatric disorders (mood, cognitive and sleep disorders, and fatigue) at the initial diagnosis and throughout the entire course of PD.

## 1. Introduction

Parkinson's disease (PD) is a progressive, complex disorder characterized by motor symptoms (bradykinesia, rigidity, resting tremor, and postural instability) as well as a wide range of nonmotor symptoms (NMS) that contribute to significant morbidity and disability [1, 2]. It is important to identify those NMS that most influence the patient's life, since they may not be evident during the clinical examination [3]. NMS like depression, anxiety, cognitive decline, pain, fatigue, insomnia, and autonomic dysfunction (constipation, urinary symptoms) are significant factors that diminish health-related quality of life (HRQoL) of individuals with PD [4–6]. While common, they are still often underrecognized in clinical practice because patients fail to spontaneously mention them, and healthcare professionals also fail to systematically ask about them [5, 7].

Nonmotor symptoms occur not only in advanced but also in early stages of PD. Some symptoms, for example, olfactory deficit, constipation, rapid-eye movement sleep behavior disorder, and depression, can even precede the appearance of motor symptoms by many years [8]. Most of the PD-associated NMS are believed to be caused by the nondopaminergic systems, in accordance with the hypothesis that other neurotransmitters including serotoninergic, noradrenergic, and cholinergic transmission are involved [9]. However, in a recent study on advanced PD subjects, Storch and coworkers [10] showed that seven out of ten selected NMS (with the exception of dysphagia, excessive sweating, and bladder urgency) fluctuated in conjunction with motor symptom fluctuations. Moreover, they were also more frequent and more severe in “off” than in the “on” state. This evidence indicates that any optimization of dopaminergic therapies must also address the management of NMS.

In light of the above facts, our study aimed to determine the impact of NMS on HRQoL in a cohort of Central European PD patients from the Slovak Republic who were already on dopaminergic medications. We hypothesized that the involvement of individual NMS domains would differ depending on the disease stage. Therefore, special emphasis was placed on a comparison of early-stage and advanced-staged PD patients.

## 2. Subjects and Methods

### 2.1. Subjects

One hundred consecutive patients with idiopathic PD diagnosed according to the UK Parkinson's Disease Society Brain Bank criteria [11] were recruited from two specialized movement disorder units of tertiary centers. All subjects gave their informed written consent in accordance with the Declaration of Helsinki, and the local Ethics Committee approved the study protocol. Patients with mild cognitive impairment were included if their informed written consent could be obtained and they were able to complete clinimetric questionnaires alone or with the help of their caregiver. All patients had been on dopaminergic therapy with levodopa (plus dopa decarboxylase inhibitor) and/or dopamine agonists for at least 3 months. Untreated subjects or those on advanced therapies like deep brain stimulation, subcutaneous apomorphine pump, or intrajejunal levodopa infusion pump were not included. For analysis the patients were divided into “early-stage” (ES) and “advanced-stage” (AS) groups on the basis of the modified Hoehn and Yahr score (H&Y) [12], the cut-off of 2.5, and a history of late complications of levodopa therapy. Six patients were classified in H&Y1, 1 in H&Y1.5, 31 in H&Y2, and 9 in H&Y2.5 (ES subjects). Forty-five people were in H&Y stage 3, 7 in H&Y4, and 1 in H&Y5 (AS patients). Dopaminergic therapy load was expressed in terms of levodopa equivalent daily dose (LEDD) [13]. Each person was in the state in which Parkinson motor symptoms were best controlled during assessment. Fluctuating patients were assessed in their optimal “on” state. Demographic and basic clinical data are shown in Table 1.

### 2.2. Methods

Validated Slovak translations of the following screening and diagnostic instruments were applied:

- the Parkinson's disease questionnaire with eight dimensions (PDQ-8), health-related quality of life measure [14], a summary index (PDQ-8 SI) which was used for statistical analysis,
- *the nonmotor symptoms screening questionnaire (NMSQuest)*, a 30-item tool, which records the presence or absence of nonmotor symptoms [15],
- *the nonmotor symptom assessment scale for Parkinson's disease (NMSS)*, a 30-item scale for assessing NMS, which covers nine dimensions: cardiovascular, sleep/fatigue, mood/apathy, perceptual problems, attention/memory, gastrointestinal, urinary, sexual function, and miscellaneous [16].

Questionnaires PDQ-8 and NMSQuest were completed by patients (with the aid of caregivers if necessary) while waiting to be seen by a neurologist. The NMSS was administered by a trained investigator.

#### 2.2.1. Statistical Analysis

Statistical examination of the data was performed using IBM SPSS Statistics 20. Clinical and demographic variables of both groups of PD patients were compared by Student's *t*-test (*P* < 0.05). The Kolmogorov-Smirnov test showed that not all data were normally distributed, so Spearman's rank correlation coefficient (Rho) was used to evaluate the association between PDQ-8 and demographic and clinical variables, NMSQuest score, NMSS total score, and nine different NMSS domains. The strength of the association for correlation coefficients was interpreted as follows: ≤0.19, negligible; 0.20 to 0.39, weak; 0.40 to 0.59, moderate; 0.60 to 0.79, strong; and ≥0.80, very strong [17].

## 3. Results

Compared to ES patients, AS subjects had significantly higher scores on the PDQ-8, NMS-Quest (number of NMS), and NMSS (Table 1). The average declared number of NMS in all 100 patients was 11.3; the average number in ES was 9.6, and in AS 12.8.

Table 2 presents the correlations found between quality of life measure PDQ-8 and selected variables of interest. The evaluation of all 100 patients showed a weak correlation with the duration of PD and NMSS cardiovascular aspects, NMSS sleep/fatigue, NMSS/perceptual problems, NMSS/gastrointestinal aspects, and NMSS/miscellaneous. The H&Y score, NMSQuest, NMSS total, NMSS mood/apathy, and NMSS attention/memory domain revealed correlations, which were moderate to strong. Similar moderate-to-strong relationships were seen in ES subjects. Moreover, there was a weak correlation with NMSS sleep/fatigue. The AS group had a PDQ-8 score that correlated moderately with NMSQuest, NMSS total, and NMSS sleep/fatigue scores. Weak correlations were found for NMSS cardiovascular, NMSS mood/apathy, and NMSS attention/memory domains.

## 4. Discussion

Although NMS greatly influence the HRQoL of PD patients, more than 50% of existing NMS are not identified in clinical practice [5].

The prevalence of overall NMS in our cohort of 100 PD subjects on dopaminergic drugs was 11/30. This is as high as reported by most other studies. [3, 5, 15, 16, 18] According to the NMSQuest (evaluation of prevalence) and NMSS (frequency × severity) the nonmotor symptoms were significantly associated with poor HRQoL across the entire clinical spectrum of PD. This finding agrees with the proposal to consider the frequency and severity of nonmotor symptoms as a whole to be the most important predictor of HRQoL in PD [3].

Surprisingly, separate evaluation of NMSS domain correlation profiles for early-stage and advanced-stage groups did not reveal any fundamental differences; however, the degree of the association with HRQoL differed. The neuropsychiatric domains attention/memory and mood/apathy correlated moderately to strongly with HRQoL in the ES group, while HRQoL was moderately related to the sleep/fatigue domain in the AS group. Other weak but still significantly correlating domains were sleep/fatigue in ES and cardiovascular aspects, attention/memory, and mood/apathy in AS. Our data are thus in line with previous studies reporting that fatigue, sleep disturbances, apathy, and mood are independent determinants of HRQoL [3–5, 19–22].

As our subjects had been optimally “tuned up” with dopaminergic therapy (according to the clinicians' impressions) and fluctuating patients were assessed in their optimal “on” state, we can assume that nondopaminergic lesions influenced their HRQoL the most.

An important limitation of our study is that the most advanced phases of disease were underrepresented. Other drawbacks are the absence of severity measures for motor symptoms, like UPDRS motor score, to determine the impact of motor system involvement, and the fact that special attention was not given to nondopaminergic add-on therapies, for example, antidepressants, hypnotics, cognitives, and laxatives.

In conclusion, our data confirm that NMS are very common and significantly influence HRQoL even in PD patients on dopaminergic therapy. These observations indicate that systematic, active screening for NMS and above all therapeutic strategies for neuropsychiatric disorders (mood, cognition, sleep, and fatigue) are urgently required both at the initial diagnosis of PD and across the entire disease course. Further studies are needed to investigate how to improve the HRQoL of PD patients and relieve their NMS.

## Figures and Tables

**Table 1:** Comparative statistics of demographic and basic clinical data and of scores of selected screening and diagnostic instruments.

	All	ES	AS	*P* value of *t*-test*
*N*	100	47	53	
Gender	50 M : 50 F	26 M : 21 F	24 M : 29 F	
Hoehn and Yahr score	2.6 ± 0.7 (1–5)	2.0 ± 0.4 (1–2.5)	3.2 ± 0.4 (3–5)	<0.01
Age (±SD); years	65.5 ± 8.8 (47–85)	62.9 ± 8.0 (47–76)	67.9 ± 8.9 (47–85)	<0.01
Disease duration; years	5.9 ± 4.4 (0.5–19)	4.1 ± 3.4 (0.5–14)	7.5 ± 4.6 (0.5–19)	<0.01
LEDD; mg	935.5 ± 476.6 (100–2000)	702.1 ± 337.0 (100–1500)	1142.5 ± 489.1 (300–2000)	<0.01
NMSQuest score^○^	11.3 ± 4.6 (0–20)	9.6 ± 4.4 (0–18)	12.8 ± 4.4 (1–20)	<0.01
NMSS total score^○^	44.2 ± 26.7 (1–170)	37.7 ± 20.5 (1–82)	50 ± 30.2 (11–170)	=0.02
PDQ-8 summary index^○^	29.3 ± 17.8 (0–69)	21.9 ± 15.0 (0–53)	35.8 ± 17.6 (0–69)	<0.01

Abbreviations: AS: advanced-stage Parkinson's disease; ES: early-stage Parkinson's disease; M: male; F: female; LEDD: levodopa equivalent daily dose.

*Comparison between early-stage and advanced-stage patients.

^○^For explanation, see Section 2.2.

Values are displayed as arithmetic mean ± standard deviation (range).

**Table 2:** Correlations expressed as Spearman's rank correlation coefficient between quality of life measure and selected variables of interest.

	PDQ-8					
	All		ES		AS	
	Rho	*P* value	Rho	*P* value	Rho	*P* value
Age	0.078	0.442	0.101	0.497	−0.056	0.688
Disease duration	0.253*	**0.011**	−0.065	0.663	0.246	0.076
LEDD	0.161	0.109	−0.177	0.234	0.076	0.589
Hoehn and Yahr score	0.447**	**<0.001**	—	—	—	—
NMSQuest^○^	0.612***	**<0.001**	0.603***	**<0.001**	0.501**	**<0.001**
NMSS total^○^	0.503**	**<0.001**	0.434**	**0.002**	0.547**	**<0.001**

NMSS domain						
Cardiovascular	0.261*	**0.009**	0.144	0.334	0.289*	**0.036**
Sleep/fatigue	0.383*	**<0.001**	0.331*	**0.023**	0.451**	**0.001**
Mood/apathy	0.474**	**<0.001**	0.604***	**<0.001**	0.348*	**0.011**
Perceptual problems	0.229*	**0.022**	0.147	0.324	0.191	0.172
Attention/memory	0.446**	**<0.001**	0.481**	**<0.001**	0.376*	**0.006**
Gastrointestinal	0.264*	**0.008**	0.248	0.092	0.256	0.064
Urinary	0.136	0.176	−0.124	0.407	0.202	0.146
Sexual function	0.146	0.147	0.089	0.554	0.268	0.053
Miscellaneous	0.253*	**0.011**	0.264	0.073	0.148	0.291

Abbreviations: AS: advanced-stage Parkinson's disease; ES: early-stage Parkinson's disease; LEDD: levodopa equivalent daily dose.

^○^For explanation, see Section 2.2.

The strength of the association for correlation coefficients is interpreted as follows: *0.20 to 0.39, weak; **0.40 to 0.59, moderate; ***0.60 to 0.79, strong; significant values are bold.
